# Impact of Lesion Preparation Technique on Side Branch Compromise in Calcified Coronary Bifurcations: A Subgroup Analysis of the PREPARE-CALC Trial

**DOI:** 10.1155/2020/9740938

**Published:** 2020-11-11

**Authors:** Abdelhakim Allali, Mohamed Abdel-Wahab, Hussein Traboulsi, Rayyan Hemetsberger, Nader Mankerious, Robert Byrne, Volker Geist, Mohamed El-Mawardy, Dmitriy Sulimov, Ralph Toelg, Gert Richardt

**Affiliations:** ^1^Cardiology Department, Heart Center Segeberger Kliniken, Bad Segeberg, Germany; ^2^Cardiology Department, Heart Center Leipzig at the University of Leipzig, Leipzig, Germany; ^3^Cardiology Department, German Heart Center, Technical University of Munich, Munich, Germany; ^4^Cardiology Department, Vivantes Wenckebach Hospital, Berlin, Germany

## Abstract

**Objectives:**

To analyze the impact of different techniques of lesion preparation of severely calcified coronary bifurcation lesions.

**Background:**

The impact of different techniques of lesion preparation of severely calcified coronary bifurcation lesions is poorly investigated.

**Methods:**

We performed an as-treated analysis on 47 calcified bifurcation lesions treated with scoring/cutting balloons (SCB) and 68 lesions treated with rotational atherectomy (RA) in the PREPARE-CALC trial. Compromised side branch (SB) as assessed in the final angiogram was the primary outcome measure and was defined as any significant stenosis, dissection, or thrombolysis in myocardial infarction flow <3.

**Results:**

True bifurcation lesions were present in 49% vs. 43% of cases in the SCB and RA groups, respectively. After stent implantation, SB balloon dilatation was necessary in around one-third of cases (36% vs. 38%; *p* = 0.82), and a two-stent technique was performed in 21.3% vs. 25% (*p* = 0.75). At the end of the procedure, the SB remained compromised in 15 lesions (32%) in the SCB group and 5 lesions (7%) in the RA group (*p* = 0.001). Large coronary dissections were more frequently observed in the SCB group (13% vs. 2%; *p* = 0.02). Postprocedural levels of cardiac biomarkers were significantly higher in patients with a compromised SB at the end of the procedure.

**Conclusions:**

In the PREPARE-CALC trial, side branch compromise was more frequently observed after lesion preparation with SCB as compared with RA. Consequently, in calcified bifurcation lesions, an upfront debulking with an RA-based strategy might optimize the result in the side branch.

## 1. Introduction

Coronary calcification is encountered in around one of five percutaneous coronary intervention (PCI) procedures, and this rate is expected to further increase in an aging PCI population [1]. Severe forms of calcification impair balloon and stent delivery and increase the likelihood of stent under expansion and malapposition [2, 3].

On the other hand, coronary bifurcation lesions account for 15–20% of PCIs [4, 5]. As both the main and side branches are interpolated in a complex bifurcation core segment [6], PCI of coronary bifurcation lesions is associated with higher periprocedural complication rates as compared to nonbifurcation lesions [7].

Side branch compromise is an early described complication during PCI of bifurcation lesions [8]. The presence of initial side branch stenosis [9] and the bifurcation angle [10] are two well-known predictors of side branch compromise. In addition, main vessel plaque can cause carina shift leading to impairment of side branch flow [11]. Plaque composition including the presence of calcification together with plaque dimensions and the degree of stenosis in the main branch have also been reported as important predictors of side branch compromise [12, 13].

In the PREPARE-CALC (the Comparison of Strategies to PREPARE Severely CALCified Coronary Lesions) randomized trial, we compared lesion preparation strategies of severely calcified coronary lesions using a scoring or cutting balloon (SCB) versus rotational atherectomy (RA) [14]. A trend was observed towards higher rates of side branch compromise in the SCB group in the total trial population. The current substudy of PREPARE-CALC aims to investigate the impact of different lesion preparation strategies of severely calcified coronary lesions located at a bifurcation segment on procedural and short-term outcomes.

## 2. Materials and Methods

### 2.1. Trial Design and Patient Population

The trial design and study population have been described in detail elsewhere [14]. Briefly, PREPARE-CALC was an investigator-initiated, randomized controlled trial enrolling patients with documented myocardial ischemia and severely calcified native coronary lesions undergoing PCI. Between September 2014 and October 2017, two hundred patients with documented myocardial ischemia and severe calcification of the target native coronary lesion as defined by cineangiography (radiopacities noted without cardiac motion before contrast injection generally compromising both sides of the arterial lumen [15]) were enrolled in two high-volume centers in Germany.

Patients were randomized 1 : 1 to an initial strategy of lesion preparation using RA versus SCB followed by implantation of a new-generation sirolimus-eluting stent with bioabsorbable polymer (Orsiro; Biotronik AG, Bülach, Switzerland). Principal exclusion criteria were myocardial infarction (MI) within 1 week, decompensated heart failure, target lesions in coronary artery bypass grafts, in-stent restenosis, and target vessel thrombus. An independent clinical events committee, blinded to treatment assignment, adjudicated all major adverse events. The study was approved by the local ethics committees of the participating centers, and each patient provided written informed consent for inclusion in the trial.

### 2.2. Bifurcation Subgroup

We performed a post hoc as-treated analysis of lesions located at a bifurcation segment. Lesions which did not involve a bifurcation were excluded. Coronary bifurcation lesions were defined as coronary artery narrowing occurring adjacent to and/or involving the origin of a significant side branch. A significant side branch was defined as having a diameter >2 mm or that supplies a significant myocardial area [16, 17]. The decision of performing lesion preparation using RA or SCB in the bifurcation (only main branch, only side branch, or both) was left to the operator's discretion.

Patients in whom a crossover from a balloon-based strategy to rotational atherectomy was performed were included in the RA group. In addition to lesions and procedural characteristics collected during the study period, two interventional cardiologists reviewed all coronary angiograms and index procedures to collect more details concerning the anatomy, the treatment steps as well as the final angiographic result of the bifurcation lesions.

Compromised side branch at the end of the procedure was the primary outcome measure of the analysis and was defined as one or more of the following: any significant stenosis (>70% diameter stenosis), dissection of the side branch, and/or final thrombolysis in myocardial infarction (TIMI) flow <3. We also compared the evolution of cardiac biomarkers between patients with a compromised side branch and those without a compromised side branch at the end of the procedure.

Baseline, postprocedural, and follow-up coronary angiograms of the target lesions of the main trial were digitally recorded and assessed off-line by the quantitative angiographic core laboratory (ISA Research Center, Munich, Germany) with an automated edge detection system (QAngio, version 7.3; Medis Medical Imaging Systems, the Netherlands) by independent personnel blinded to treatment allocation [14]. Results concerning blinded QCA of target lesions involving a bifurcation are reported in this analysis.

### 2.3. Statistical Methods

Statistical analysis was performed using Stata SE 14 (StataCorp LP, Texas, USA). Quantitative variables are summarized as mean ± SD or median (interquartile range (IQR)) and are compared by the 2-sided unpaired *t*-test. Categorical variables are summarized as frequencies and proportions and are compared by the chi-square or Fisher's exact tests. All probability values were two-tailed, and a *p* value of <0.05 was considered significant.

## 3. Results

### 3.1. Study Population

**Figure 1:**
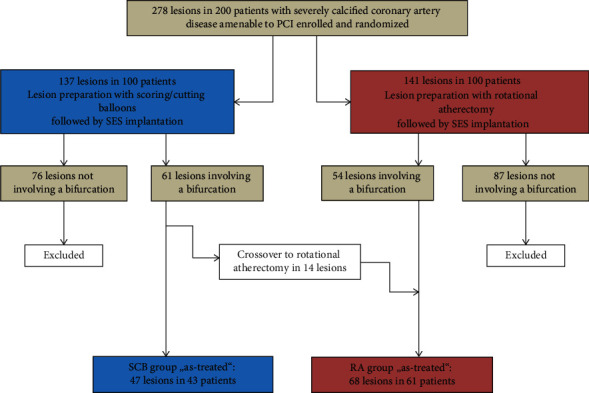
The study flowchart. SCB=scoring/cutting balloons; PCI=percutaneous coronary intervention; RA=rotational atherectomy.

The study flowchart is illustrated in Figure 1, and baseline characteristics are summarized in Table 1. Of 278 lesions in the 200 patients enrolled in the PREPARE-CALC trial, we identified two study groups for the present analysis: the SCB group including 47 bifurcation lesions in 43 patients (mean age 75.0 ± 6.6 years; 79% males) and the RA group with 68 lesions located at bifurcations in 61 patients (mean age 74.8 ± 6.5 years, 77% males). In the RA group, 11 patients with 14 lesions had been initially randomized to an SCB strategy, but during the index procedure bailout RA was required. More patients in the SCB group had a history of previous myocardial infarction compared to the RA group (39% vs. 16%; *p* = 0.008). Other clinical characteristics were balanced between both groups.

### 3.2. Angiographic and Procedural Characteristics

Angiographic and procedural characteristics are shown in Table 2. Lesions were mostly located in the LAD (60% in the SCB group and 63% in the RA group). Side branches were larger than 2 mm in 41 lesions (87%) in the SCB group and 54 lesions (79%) in the RA group; the angle between the side and main branch was most commonly less than 70° (62% vs. 66%; *p* = 0.62). Lesions were mostly classified as “1.1.0” and “1.1.1” according to the Medina classification (80.9% of lesions). Although a true bifurcation lesion (Medina *x*.*x*.1) was described in 49% of lesions in the SCB group and 43% in the RA group (*p* = 0.50), lesion preparation of the side branch with SCB or RA was performed in only 6.3% and 2.9%, respectively (*p* = 0.38).

A single-stent technique was used in most lesions (68% vs. 75%; *p* = 0.89), and in around one-third of cases, an intervention in the side branch with balloon dilatation or kissing-balloon inflation was necessary (36% vs. 38%; *p* = 0.82). Proximal optimization technique (POT) was performed in 60% and 62% among SCB and RA groups, respectively. Culotte (12 lesions) and T-and-protrusion (9 lesions) stenting were the most frequently performed two-stent techniques. The number of implanted stents per lesion was higher in the SCB group (1.85 ± 0.92 vs. 1.50 ± 0.68; *p* = 0.02).

A compromised side branch at any time during the procedure was documented in 53% of lesions in the SCB group and 41% in the RA group (*p* = 0.20). At the end of the procedure, residual side branch compromise—the primary outcome measure of this study—was observed in 15 lesions (32%) in the SCB group and in 5 lesions (7%) in the RA group (*p* = 0.001).

Details of blinded core lab quantitative coronary angiographic analysis of the target lesions involving bifurcations are listed in Table S1. In general, angiographic characteristics of treated lesions, acute lumen gain after the procedure, and late lumen loss at 9 months were not significantly different between the two groups.

### 3.3. Procedural and In-Hospital Events

Procedural and in-hospital events are shown in Table 3. Fluoroscopy time and procedural duration were significantly higher in the RA group. Large coronary dissections were more frequently observed in the SCB group (13% vs. 2%; *p* = 0.02). In-hospital outcome was similar between both groups. Protocol-defined periprocedural myocardial infarction occurred in only one patient in the RA group.

**Figure 2:**
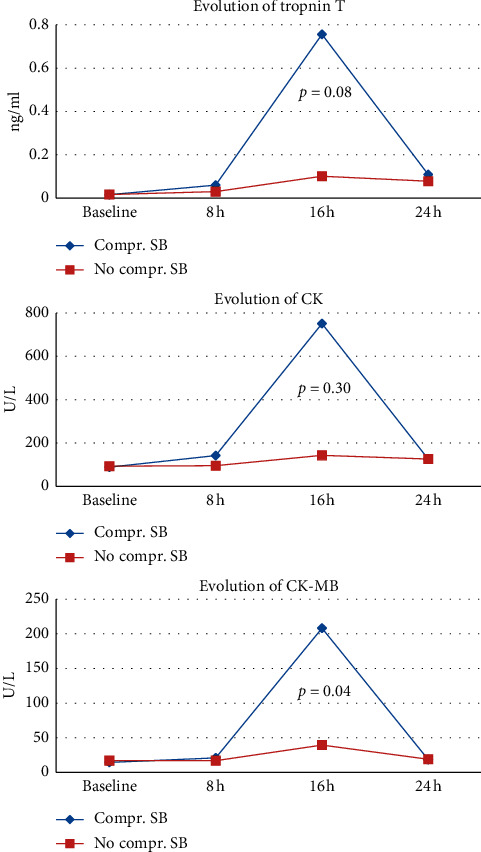
Evolution of median value of cardiac biomarkers in patients with (*n*=20) and without (*n*=84) compromised side branch at the end of the procedure.

We further analyzed the evolution of cardiac biomarkers in patients with (*n* = 20) and without (*n* = 84) a compromised side branch at the end of the procedure until 24 hours postprocedure. The median value of CK-MB was significantly higher at 16 hours post-PCI in patients with a compromised side branch (208 U/L vs. 39.5 UL; *p* = 0.04) with a trend towards higher troponin T levels (0.756 ng/ml vs. 0.101; *p* = 0.08) (Figure 2).

## 4. Discussion

The main findings of this study can be summarized as follows:

- Comparing a strategy of balloon dilatation using a scoring/cutting balloon vs. an atheroablative method using rotational atherectomy in the main vessel of calcified lesions involving a bifurcation, the atheroablative strategy resulted in fewer compromised side branches at the end of the procedure
- Myocardial injury, as assessed by cardiac markers of necrosis was higher in patients with a compromised side branch at the end of the procedure

Treatment of bifurcation lesions is a growing challenge in contemporary PCI because of the technical complexity, higher risk of periprocedural complications, and worse outcomes compared to nonbifurcation lesions [18]. In addition to the difficulties related to lesion preparation and device delivery, the presence of coronary calcification represents an additional challenge in treating bifurcation lesions. Fujino et al. demonstrated that the presence of coronary calcification at the site of a bifurcation assessed by optical coherence tomography is associated with a higher risk of side branch occlusion [19]. In this context, the main issues to provide optimal vessel patency are careful lesion preparation to “soften” the lesion, prevention of plaque shifting, and careful carina reconstruction [20].

In the single-arm prospective AGILITY (AngioSculpt Coronary Bifurcation Study) trial [21], the scoring balloon was used for the side branch dilatation prior to the deployment of a drug-eluting stent in the main vessel as a modified provisional strategy in true bifurcation lesions. In that study, the postscoring balloon dissection rate was 6% in the side branch and crossover to stent deployment of the affected side branch was required in only 10.8%. Despite the promising findings of this study, moderate to severe calcification was only present in 24.7% of the main vessel, whereas in the PREPARE-CALC trial severe coronary calcification was the main inclusion criterion. Furthermore, lesion preparation in our study was mainly performed in the main vessel rather than the side branch.

Several observational studies support the safety and effectiveness of RA in calcified bifurcation lesions, as high (>90%) success rates can be achieved, and the need for bailout side branch stenting is less than 20% [22, 23]. Recently, Chambers et al. demonstrated in a series of patients undergoing lesion preparation with either orbital atherectomy or RA for severely calcified lesions similar low 30-day MACE rates when comparing patients with bifurcation versus nonbifurcation lesions [24].

Local factors such as hemodynamic forces play a major role in the formation of atherosclerosis and its regional distribution. Atherosclerosis has a predilection for the outer walls of bifurcations, where shear stress is lower and blood flow is turbulent, whereas the carina is typically spared from plaque precipitation. The mechanism of side branch closure due to calcium has not been fully elucidated, but a higher risk of carina shift due to reduced compliance of the wall opposing the side branch and a lower resistance encountered by the inflated balloon at the SB ostium could be a mechanism [25]. The differences in the mechanism of lesion preparation could therefore explain the superiority of RA over SCB in preventing side branch compromise observed in our study. Rotational atherectomy ablates hard plaque components while displacing and sparing soft tissues by the so-called differential cutting principle [26]. Although in current practice the role of RA has changed from aggressive debulking to plaque modification [27], the atheroablative effect of RA by reducing the volume of the plaque might still reduce the risk of carina shift during lesion preparation and after stent implantation.

Cutting and scoring balloons are designed to cut or fracture the continuity of fibrocalcific plaques without any atheroablative effect. Therefore, the plaque compression during balloon inflation could result in carina shift and SB disturbance [28]. Moreover, in our analysis, we observed higher rates of coronary dissection in the SCB group (13% vs. 2%; *p* = 0.02). The extension of coronary dissection from the treated main branch to the side branch might explain the more frequent side branch dissection and slow flow observed in that group.

Although more compromised side branches were observed in the SCB group, the rate of SB stenting was not significantly different between the groups. This finding could be related to the higher number of stents implanted per lesion in the SCB group. Nevertheless, the analysis of cardiac biomarkers revealed that myocardial injury following PCI was more frequent in patients with a compromised side branch at the end of the procedure compared with those without SB compromise. Garcia-Garcia et al. showed in a recent analysis of pooled data from five coronary stent trials and one large registry that CK-MB elevation after PCI was independently associated with mortality at one year [29].

### 4.1. Study Limitations

The PREPARE-CALC trial was powered to assess strategy success of SCB versus RA followed by DES implantation in calcified coronary lesions. The current analysis is a post hoc analysis consisting of about half of the patients originally enrolled in this trial; the results should therefore be considered hypothesis-generating as the groups were not adequately powered to detect a difference.

Another limitation arises from the definition of “significant side branch” which was based upon a subjective judgment of the operator as a branch that the operator does not want to lose. In addition, a dedicated bifurcation QCA was not performed. Compared to the high number of true bifurcation lesions, lesion preparation using SCB or RA in the side branch was performed in a very limited number of lesions (5 lesions overall) and this could affect the outcome in case of severe calcification in the side branch.

Finally, this analysis suffers from the same limitations as previously described for the PREPARE-CALC trial, i.e., the majority of cases in the SCB group were performed using scoring rather than cutting balloons. Patients with only stable coronary artery disease were included.

## 5. Conclusion

Comparing a strategy of SCB versus RA in severely calcified coronary bifurcation lesions, we observed a significantly higher rate of side branch compromise with an SCB-based strategy, which did not translate into worse short-term clinical outcome in this small cohort. Side branch compromise was associated with more extensive periprocedural myocardial injury. Therefore, in calcified bifurcation lesions, an upfront debulking with an RA-based strategy might optimize the result of PCI in the side branch.

## Figures and Tables

**Table 1:** Baseline characteristics (*n* = 104 patients).

	SCB (*n* = 43)	RA (*n* = 61)	*p* value
Age (years)	75.0 ± 6.6	74.8 ± 6.5	0.88
Males	34 (79%)	47 (77%)	0.81
Height (cm)	173.9 ± 8.7	171.6 ± 8.9	0.20
Weight (kg)	83.0 ± 13.0	82.3 ± 16.1	0.83
Diabetes mellitus	9 (21%)	21 (34%)	0.13
Hypertension	39 (91%)	58 (96%)	0.38
Dyslipidemia	26 (60%)	45 (74%)	0.15
Current smokers	6 (13%)	9 (15%)	0.91
Chronic renal failure^*∗*^	11 (26%)	12 (20%)	0.50
Previous MI	17 (39%)	10 (16%)	0.008
Previous PCI	20 (46%)	26 (43%)	0.69
Previous CABG	6 (14%)	6 (10%)	0.52
Unstable angina	2 (5%)	4 (7%)	0.68
Atrial fibrillation	3 (7%)	11 (18%)	0.10
Left main disease	21 (49%)	25 (41%)	0.43
Multivessel disease	38 (88%)	54 (88%)	0.98
LV ejection fraction (%)	57.5 ± 10.3	57.9 ± 10.1	0.86
Multilesion PCI	22 (51%)	29 (48%)	0.72
Unfractionated heparin	43 (100%)	61 (100%)	1.00
Bivalirudin	0 (0%)	0 (0%)	1.00
GP IIb/IIIa antagonists	0 (0%)	2 (3%)	0.51

Values are *n* (%) or mean ± SD; CABG=coronary artery bypass graft, GP=glycoprotein, LV=left ventricle, MI=myocardial infarction, PCI=percutaneous coronary intervention, RA=rotational atherectomy, and SCB=scoring/cutting balloon. ^*∗*^Glomerular filtration rate <60 ml/min.

**Table 2:** Angiographic and procedural characteristics (*n* = 115 lesions).

	SCB (*n* = 47)	RA (*n* = 68)	*p* value
*Angiographic characteristics*			
Location			0.84
Left main	12 (25.5%)	15 (22.1%)	
Left anterior descending	28 (59.6%)	43 (63.2%)	
Left circumflex	5 (10.6%)	8 (11.8%)	
Right coronary artery	2 (4.3%)	2 (2.9%)	
Reference vessel diameter (mm)	3.27 ± 0.46	3.26 ± 0.45	0.87
Lesion length (mm)	26.06 ± 13.84	27.34 ± 14.83	0.64
Diameter stenosis (%)	83.57 ± 8.99	85.94 ± 9.63	0.19
Ostial location	15 (31.9%)	31 (45.6%)	0.14
Angle > 70% between MB and SB	18 (38.3%)	23 (33.8%)	0.62
SB > 2 mm	41 (87.2%)	54 (79.4%)	0.28
True bifurcation lesions^*∗*^	23 (49%)	29 (43%)	0.50
Medina classification			
1.1.0	19 (40.4%)	30 (44.1%)	
1.1.1	18 (38.3)	26 (38.2%)	
1.0.1	2 (4.2%)	1 (1.5%)	
1.0.0	0	1 (1.5%)	
0.1.1	2 (4.2%)	1 (1.5%)	
0.1.0	5 (10.6%)	8 (11.7%)	
0.0.1	1 (2.1%)	1 (1.5%)	

*Procedural characteristics*			
SCB or RA in SB	3 (6.3%)	2 (2.9%)	0.38
Wire in SB	28 (59.7%)	36 (52.9%)	0.48
SB kissing or balloon dilation after stenting	17 (36.2%)	26 (38.2%)	0.82
Stenting technique			0.75
One-stent technique	36 (76.6%)	51 (75%)	
Two-stent technique	10 (21.3%)	17 (25%)	
Culotte	6 (12.8%)	6 (8.8%)	
T-stenting	1 (2.1%)	2 (2.9%)	
DK-crush	0	1 (1.5%)	
Minicrush	1 (2.1%)	1 (1.5%)	
TAP stenting	2 (4.2%)	7 (10.3%)	
No stent	1 (2.1%)	0	
Stenting of the SB	10 (21.3%)	17 (25%)	0.85
Elective	7 (14.9%)	11 (16.2%)	
Bailout	3 (6.4%)	6 (8.8%)	
POT	28 (59.6%)	42 (61.8%)	0.81
LMT involvement during the PCI	21 (44.7%)	28 (41.2%)	0.71
Cutting/scoring balloon diameter (mm)	3.04 ± 0.32	—	—
Cutting/scoring balloon pressure (atm)	15.30 ± 2.3	—	—
Starting burr size (mm)	—	1.51 ± 0.17	—
Max. burr size (mm)	—	1.53 ± 0.17	—
Use of >1 burr	—	6 (8.8%)	-—
Rotational speed (RPM)	—	164,895 ± 22,038	—
Number of predilatation balloons	1.74 ± 0.85	1.75 ± 0.98	0.95
Max. predilatation balloon diameter (mm)	2.78 ± 0.47	2.90 ± 0.38	0.16
Max. predilatation balloon pressure (atm)	18.22 ± 3.43	19.50 ± 5.08	0.20
No. of stents/lesions	1.85 ± 0.92	1.50 ± 0.68	0.02
Total stent length/lesion (mm)	37.09 ± 17.63	33.88 ± 15.71	0.31
Min. stent diameter (mm)	3.14 ± 0.41	3.08 ± 0.47	0.50
Max. stent diameter (mm)	3.43 ± 0.41	3.30 ± 0.40	0.09
Max. stent implantation pressure (atm)	16.61 ± 2.83	16.92 ± 3.32	0.57
Balloon postdilatation	41 (87.2%)	62 (91.1%)	0.50
Max. postdilatation balloon diameter (mm)	3.56 ± 0.56	3.65 ± 0.53	0.06
Max. postdilatation balloon pressure (atm)	20.54 ± 3.23	21.13 ± 4.10	0.44
Procedural result on SB			
Compromised at any time during the procedure	25 (53.2%)	28 (41.2%)	0.20
Compromised at the end of the procedure	15 (31.9%)	5 (7.4%)	0.001
Mechanism of SB compromise			
Significant stenosis	13 (27.7%)	4 (5.9%)	
Dissection	2 (4.3%)	1 (1.5%)	
TIMI flow <3 in SB	4 (8.5%)	2 (3%)	

Values are *n* (%) or mean ± SD; DK-crush = double kissing crush; MB = main branch; LMT = left main trunk; POT = proximal optimization technique; RPM = rotations per minute; RA = rotational atherectomy; SB = side branch; SCB = scoring/cutting balloon; TAP = T-and-protrusion technique. ^*∗*^True bifurcation lesions: Medina 1.1.1, 1.0.1, and 0.1.1.

**Table 3:** Procedural and in-hospital outcome (*n* = 104 patients).

	SCB (*n* = 43)	RA (*n* = 61)	*p* value
Procedural duration (min)	77.9 ± 46.3	96.5 ± 36.3	0.02
Fluoroscopy time (min)	19.4 ± 15.2	26.9 ± 12.5	0.01
Contrast amount (ml)	234.3 ± 107.1	273.0 ± 116.1	0.08
Large dissection (>5 mm)	6 (13%)	1 (2%)	0.02
Perforation	2 (5%)	2 (3%)	1.00
Pericardial effusion	0 (0%)	2 (3%)	0.51
No/slow flow	0 (0%)	2 (2%)	0.49
Final TIMI flow < III in MB	0 (0%)	1 (2%)	0.41
Residual stenosis > 20% in MB	2 (5%)	0 (0%)	0.17
Stent failure^*∗*^	0 (0%)	4 (7%)	0.14
Crossover from SCB to RA^*∗∗*^	0 (0%)	11 (18%)	0.002
Death	0 (0%)	0 (0%)	1.00
Myocardial infarction	0 (0%)	1 (2%)	1.00
Target vessel re-PCI	0 (0%)	0 (0%)	1.00
CABG	0 (0%)	0 (0%)	1.00
Stent thrombosis	0 (0%)	0 (0%)	1.00
Access site complications	2 (5%)	2 (3%)	1.00

Values are *n* (%) or mean ± SD; CABG = coronary artery bypass graft; MB = main branch; PCI = percutaneous coronary intervention; RA = rotational atherectomy; SCB = scoring/cutting balloon; TIMI = thrombolysis in myocardial infarction. ^*∗*^Stent failure occurred in patients initially randomized to an SCB strategy, and a crossover to RA was performed. ^*∗∗*^The interpretation of this finding is biased by the fact that crossover patients who were initially randomized to an SCB strategy are included in the RA group.

## Data Availability

The data used to support the findings of this study may be released upon application to the steering committee of the PREPARE-CALC trial, who can be contacted at Heart Center Segeberger Kliniken GmbH, Am Kurpark 1, 23795 Bad Segeberg, Germany.

## Abbreviations

PREPARE-CALC trial: The Comparison of Strategies to PREPARE Severely CALCified Coronary Lesions
RA: Rotational atherectomy
SB: Side branch
SCB: Scoring/cutting balloons.

## Supplementary Materials

Supplementary Materials Table S1: corelab assessed quantitative coronary angiography data (*n* = 115 lesions).
