# Supplementary material for: Impact of Lesion Preparation Technique on Side Branch Compromise in Calcified Coronary Bifurcations: A Subgroup Analysis of the PREPARE-CALC Trial
Source: J Interv Cardiol. 2020 Nov 11;2020:9740938. doi: 10.1155/2020/9740938 (PMC7673940; doi:10.1155/2020/9740938)
Supplement: Supplementary Materials — Table S1: corelab assessed quantitative coronary angiography data (n = 115 lesions). [file 9740938.f1.docx]

**Table S1: Corelab assessed quantitative coronary angiography data (n=115 lesions)**

|  | **Modified balloon  (n = 47)** | **Rotational atherectomy (n = 68)** | **p-value** |
| --- | --- | --- | --- |
| **Before procedure** |  |  |  |
| **Lesion length (mm)** | 19.44±10.09 | 17.56±11.43 | 0.36 |
| **Reference vessel diameter (mm)** | 3.25±0.46 | 3.26±0.43 | 0.96 |
| **Minimal lumen diameter (mm)** | 1.08±0.34 | 1.12±0.37 | 0.57 |
| **Diameter stenosis (%)** | 65.59±9.41 | 64.61±10.96 | 0.62 |
| **Severe calcification*** | 100 (72.9%) | 104 (76.4%) | 0.46 |
| **Immediately after procedure** |  |  |  |
| **Minimal lumen diameter (mm)** |  |  |  |
| **In-stent** | 2.83±0.39 | 2.83±0.41 | 0.97 |
| **In-segment** | 2.60±0.54 | 2.51±0.54 | 0.40 |
| **Diameter stenosis (%)** |  |  |  |
| **In-stent** | 13.55±4.74 | 12.95±6.22 | 0.58 |
| **In-segment** | 17.05±7.81 | 17.63±7.90 | 0.70 |
| **Acute gain (mm)** |  |  |  |
| **In-stent** | 1.74±0.37 | 1.71±0.45 | 0.74 |
| **In-segment** | 1.51±0.54 | 1.40±0.49 | 0.26 |
| **Re-angiography at 9 Months** |  |  |  |
| **Late lumen loss**  **In-stent**  **In-segment** | 0.09±0.36  0.004±0.56 | 0.18±0.31  -0.014±0.45 | 0.21  0.86 |

Values are n (%) or mean ± SD

*as adjudicated by the angiographic corelab
